# Is HER2 ultra-low breast cancer different from HER2 null or HER2 low breast cancer? A study of 1363 patients

**DOI:** 10.1007/s10549-023-07079-8

**Published:** 2023-08-28

**Authors:** Zhaoxu Chen, Huiqing Jia, Huina Zhang, Lifang Chen, Peng Zhao, Jing Zhao, Guangming Fu, Xiaoming Xing, Yujun Li, Chengqin Wang

**Affiliations:** 1 Department of Pathology, The Affiliated Hospital of Qingdao University, No. 16 Jiangsu Road, Qingdao, 266000 Shandong China; 2 Department of Pathology, School of Basic Medicine, Qingdao University, Qingdao, 266021 Shandong China; 3 Department of Pathology, University of Rochester Medical Center, Rochester, NY 14624 USA

**Keywords:** Breast cancer, Human epidermal growth factor receptor 2(HER2), HER2 ultra-low, HER2-low, HER2-null, Clinicopathological features, Disease- free survival

## Abstract

**Objective:**

This study aims to analyze whether there are any differences in clinicopathological features and prognosis between HER2 ultra-low, HER2-null, and HER2-low expression in Chinese breast cancer (BC) patients.

**Methods:**

The clinicopathological data of 1363 HER2-negative BC patients were retrospectively collected (from January 2018 to December 2019). HER2 status was further classified into HER2-null, HER2 ultra-low, and HER2-low. HER2-null expression is defined as infiltrating cancer cells completely free of staining. HER2 ultra-low expression is defined as ≤10% of infiltrating cancer cells showing incomplete and faint/weak membrane staining. HER2-low expression is defined as HER2 immunohistochemistry (IHC) 1+ or 2+ with negative in situ hybridization (ISH) assay.

**Results:**

Of 1363 patients, there were 86 (6.3%) HER2-null patients, 395 (29.0%) HER2 ultra-low patients, and 882 (64.7%) HER2-low patients. HER2 ultra-low patients were different from HER2-low patients in terms of N stage, hormone receptor (HR) status, Ki-67 expression, and type of surgery. There were also significant differences in histologic type and postoperative endocrine therapy between HER2 ultra-low and HER2-null patients. HR+ (81.0%) tumors was more common than HR− (19.0%) in HER2 ultra-low patients. In addition, there was a significant difference in HR status between HER2 ultra-low and HER2-low patients (*P* = 0.001). The survival analysis showed that HER2 status had no effect on disease-free survival (DFS) in HER2-negative patients (all *P* > 0.05). However, regardless of HER2 status, HR+ patients had better DFS than HR− patients (*P* = 0.003). Cox multivariate analysis revealed that age (HR [95% CI] = 0.950 [0.928, 0.972], *P* < 0.001), HR status (HR [95% CI] = 3.342 [1.658, 6.736], *P* = 0.001), and postoperative endocrine therapy (HR [95% CI] = 0.048 [0.048, 0.023], *P* < 0.001) were important influencing factors of DFS in HER2-negative BC patients.

**Conclusion:**

HER2 ultra-low BC patients demonstrated distinct clinicopathological features from HER2-null and HER2-low tumors; while, HER2 status (null, ultra-low, or low) had no prognostic value in these HER2-negative BC population. Consistent with the published literature, HR status was an independent prognostic factor for DFS in HER2-negative BC patients.

## Introduction

Breast cancer (BC) has become the most common malignant tumor in women worldwide and the no. 1 malignancy that threatens the health of women in China [1]. Approximately 15% of BC patients have human epidermal growth factor receptor 2 (HER2)-positive expression, and the majority of BCs are HER2-negative by the traditional binary HER2 positive and negative classification [2–4]. Based on the American Society of Clinical Oncology (ASCO)/College of American Pathologists (CAP) HER2 testing guidelines [2] and earlier studies [5], it has shown that HER2-negative BCs have varying degrees of HER2 protein expression on the tumor cell membrane. Meanwhile, recent clinical trial studies have shown that some novel anti-HER2 therapeutic agents, especially new generation antibody–drug conjugates (ADCs) [6–9], achieved an objective response rate (ORR) of approximately 40% in patients with HER2-low expressing metastatic BC (BCs with HER2 IHC 1+ or 2+/ISH negative) [10]. This has led to a surge in research on HER2-low expressing BC. Recently, the preliminary result from DAISY trial showed that patients with HER2 ultra-low expression (BCs with ≤10% of infiltrating cancer cells showing incomplete and faint/weak membrane staining) still showed an ORR of 30.6% [11]. All these findings are challenging the traditional dichotomy of HER2 status and are constantly refreshing our understanding of the field. Nevertheless, a more comprehensive assessment of HER2 status in these tumors and a detailed description of their clinicopathological features and prognosis are still needed.

The aim of this study is to investigate the clinicopathological features and prognosis in patients with HER2 ultra-low, HER2-null, and HER2-low expression in the Chinese BC population, and hopefully to provide more insights in these HER2-negative BCs.

## Materials and methods

### Patients and clinicopathological data

In this retrospective study, we included the clinicopathological data of 1363 female patients with primary invasive BC who did not receive neoadjuvant chemotherapy in the Affiliated Hospital of Qingdao University between January 2018 and December 2019. Patients with concurrent bilateral BC (bilateral tumors diagnosed <6 months apart), combined with other malignancies, or treated with neoadjuvant chemotherapy were excluded. Clinicopathological information [including age, menopausal status, breast tumor location, tumor size, tumor multifocality, histologic type, histologic grade, TNM-staging, hormone receptor (HR) status, Ki-67, tumor infiltrating lymphocytes (TILs), type of surgery] was collected from clinical medical records and pathology databases. Disease-free survival (DFS) is the time interval from surgery to disease progression (including ipsilateral or contralateral BC recurrence, local/distant metastases) or death in BC patients. Follow-up was concluded in April 2023. This study was approved by the Ethics Committee of the Affiliated Hospital of Qingdao University(QYFY WZLL 27903).

### Assessment of HER2 expression

All cases were reviewed and retrieved by two pathologists and the assessment of HER2 status was performed according to the latest guideline of ASCO and/or CAP [2]. HER2 protein was determined by IHC (clone 4B5, prediluted, Ventana), and HER2 gene amplification was evaluated by fluorescence in situ hybridization (FISH, Ventana). HER2-null expression is defined as infiltrating cancer cells completely free of staining. HER2 ultra-low expression is defined as ≤10% of infiltrating cancer cells showing incomplete and faint/weak membrane staining. HER2-low expression is defined as HER2 IHC 1+ or 2+ with negative ISH assay.

### Statistical analysis

The clinicopathological characteristics of BC patients with null, ultra-low, and low HER2 expression (SPSS 26.0, IBM, USA) were analyzed using the Chi-square test or Fisher’s exact test for categorical variables and *T* test for continuous variables. Kaplan–Meier curve was used to describe the DFS, and log–rank test was used for comparison (GraphPad Prism 9.0,USA). In multivariate analysis, Cox proportional hazards model was used to assess the correlation between survival outcomes and potential prognostic factors based on hazard ratios (HR) and 95% confidence intervals (CI). For all statistical analysis, *P* value of <0.05 is considered statistically significant.

## Results

### Population description and follow up

Of 1363 patients, there were 86 (6.3%) HER2-null patients, 395 (29.0%) HER2 ultra-low patients, and 882 (64.7%) HER2-low patients. The median age of the patients in the entire cohort was 52 years old (range 24–89 years) and the mean age was 53.2 years old. The sizes of the BC tumor were 0.3–10 cm (median 2 cm). 91.0% patients had single focus of tumor and 9.0% had multiple foci of tumors, and the most common histologic type was ductal (84.0%). Most patients had intermediate (68.2%) or poorly (25.9%) differentiated tumors. The most common surgery type was mastectomy + axillary lymph node dissection (44.2%). 62.1% of patients received postoperative chemotherapy therapy, whereas only 20.3% of patients received postoperative radiation therapy. With a median follow-up of 53 months, 55 cases presented with recurrence or distant metastases, and 17 cases lost to follow-up. At the end of the follow-up, no deaths occurred.

### Correlation between HER2 expression and clinicopathological features

Comparing the clinicopathological features (Table 1), HER2 ultra-low patients were different from HER2-low patients in terms of N stage (*P* = 0.048), HR status (*P* = 0.001), Ki-67 expression (*P* < 0.001), and type of surgery (*P* = 0.049). There were also significant differences in histologic type (*P* = 0.016) and postoperative endocrine therapy (*P* = 0.020) between HER2 ultra-low and HER2-null patients. No differences were found in age, menopausal status, tumor location, size, multiplicity, grade, T stage, TILs density, recurrence or metastasis, postoperative chemotherapy, radiotherapy, and endocrine therapy (all *P* > 0.05).

**Table 1:** Baseline clinicopathological characteristics according to HER2 status of 1363 BC patients

Clinicopathological characteristics	Overall (*N* = 1363, %)	HER2-null (86, 6.3%)	HER2 ultra-low (395, 29.0%)	HER2-low (882, 64.7%)	P (HER2 status)	
					Null vs ultra-low	Ultra-low vs low
Age/years						
Median/range	52/24–89	52/30–79	51/24–85	53/25–89	0.791*	0.129*
Mean ± SD	53.2 ± 11.3	52.8 ± 11.4	52.5 ± 11.1	53.5 ± 11.3		
Menopaual status						
Pre/perimenopausal	771 (56.6)	47 (54.7)	232 (58.7)	492 (55.8)	0.487	0.325
Postmenopausal	592 (43.4)	39 (45.3)	163 (41.3)	390 (44.2)		
Breast position						
Left	696 (51.1)	44 (51.2)	206 (52.2)	446 (50.6)	0.868	0.600
Right	667 (48.9)	42 (48.8)	189 (47.8)	436 (49.4)		
Tumor size/cm						
Median/range	2/0.3–10	2/0.6–5.5	1.9/0.4–7.5	2/0.3–10	0.982*	0.401*
Mean ± SD	2.2 ± 1.1	2.1 ± 0.7	2.1 ± 1.0	2.2 ± 1.1		
Tumor multiplicity						
Single	1240 (91.0)	79 (91.9)	361 (91.4)	800 (90.7)	0.888	0.692
Multiple	123 (9.0)	7 (8.1)	34 (8.6)	82 (9.3)		
Histologic type						
Ductal	1145 (84.0)	63 (73.3)	331 (83.8)	751 (85.1)	**0.016**	0.817
Lobular	63 (4.6)	10 (11.6)	17 (4.3)	36 (4.1)		
Other	155 (11.4)	13 (15.1)	47 (11.9)	95 (10.8)		
Grade						
1	81 (5.9)	7 (8.1)	26 (6.6)	48 (5.4)	0.057	0.066
2	929 (68.2)	44 (51.2)	256 (64.8)	629 (71.3)		
3	353 (25.9)	35 (40.7)	113 (28.6)	205 (23.2)		
pT						
1	746 (54.7)	47 (54.7)	224 (56.7)	475 (53.9)	0.787	0.600
2	586 (43.0)	38 (44.2)	163 (41.3)	385 (43.7)		
3	31 (2.3)	1 (1.1)	8 (2.0)	22 (2.4)		
pN						
0	184 (13.5)	13 (15.1)	54 (13.7)	117 (13.3)	0.261	**0.048**
1	265 (19.4)	12 (14.0)	62 (15.7)	191 (21.7)		
2	85 (6.2)	9 (10.5)	18 (4.5)	58 (6.6)		
3	51 (3.7)	2 (2.3)	15 (3.8)	34 (3.8)		
Unknown	778 (57.1)	50 (58.1)	246 (62.3)	482 (54.6)		
HR status						
Positive	1156 (84.8)	62 (72.1)	320 (81.0)	774 (87.8)	0.064	**0.001**
Negative	207 (15.2)	24 (27.9)	75 (19.0)	108 (12.2)		
Ki67						
Median/range	2/1–90	2/1–90	2/1–90	2/1–80	0.722*	<0.001*
Mean ± SD	11.5 ± 18.9	16.5 ± 24.8	15.6 ± 22.4	9.2 ± 15.8		
TILs						
Median/range	10/1–90	5/1–80	5/1–80	10/1–90	0.677*	0.517*
Mean ± SD	13.6 ± 15.0	14.0 ± 19.0	13.2 ± 14.9	13.7 ± 14.7		
Unknown	18	2	6	10		
Type of surgery						
BCS/+BR	343 (25.2)	19 (22.1)	104 (26.3)	220 (24.9)	0.717	**0.049**
Mastectomy	418 (30.6)	31 (36.0)	135 (34.2)	252 (28.6)		
Mastectomy + ALND	602 (44.2)	36 (41.9)	156 (39.5)	410 (46.5)		
Recurrence or metastasis						
Yes	55 (4.0)	6 (7.0)	13 (3.3)	36 (4.1)	0.112	0.497
No	1308 (96.0)	80 (93.0)	382 (96.7)	846 (95.9)		
Postoperative chemotherapy						
Yes	847 (62.1)	61 (70.9)	251 (63.5)	535 (60.7)	0.141	0.456
No	507 (37.2)	23 (26.8)	141 (35.7)	343 (38.9)		
Unknown	9 (0.7)	2 (2.3)	3 (0.8)	4 (0.4)		
Postoperative radiotherapy						
Yes	276 (20.3)	19 (22.1)	70 (17.7)	187 (21.2)	0.571	0.102
No	1070 (78.5)	66 (76.7)	317 (80.3)	687 (77.9)		
Unknown	17 (1.2)	1 (1.2)	8 (2.0)	8 (0.9)		
Postoperative endocrine therapy						
Yes	980 (71.9)	49 (57.0)	273 (69.1)	658 (74.6)	**0.020**	0.061
No	352 (25.8)	37 (43.0)	114 (28.9)	201 (22.8)		
Unknown	31 (2.3)	0 (0.0)	8 (2.0)	23 (2.6)		

*BC* breast cancer; *HR* hormone receptor; *TILS* tumor infiltrating lymphocytes; *BCS* breast-conserving surgery; *BR* breast reconstruction; *ALND* axillary lymph node dissection; *SD* standard deviation

Bold indicates *P* < 0.05

*T-test; other testing methods are Chi-square test

### HER2 and HR status

The proportion of HR+ in HER2-negative tumors (1156, 84.8%) was much higher than that in HR− tumors (207, 15.2%), and HR+ (320, 81.0%) tumors was more common than HR− (75, 19.0%) in HER2 ultra-low BC patients. HR− expression was more common in HER2 ultra-low tumors; while, HR+ expression was more common in HER2-low tumors (*P* = 0.001). However, no difference in HR status was found between HER2 ultra-low and HER2-null patients (*P* = 0.064). We further analyzed the correlation between different HER2 protein expression status and HR status in HER2-negative tumors. In HR+ group, HER2 ultra-low patients had higher Ki-67 expression (*P* < 0.001), lower ER expression (*P* = 0.036), and TILs density (*P* = 0.035) than HER2-low patients. Lobular cancer (*P* = 0.004) was more common in HER2-null than in HER2 ultra-low. In the HR− group, HER2 ultra-low patients were younger (*P* = 0.013), with higher grade (*P* = 0.035) and Ki-67 expression (*P* = 0.003) than HER2-low patients, but there was no significant difference compared to HER2-null patients. The details are provided in Tables 2 and 3.

**Table 2:** Baseline clinicopathological characteristics according to HER2 status of 1156 HR+ BC patients

Clinicopathological characteristics	Overall (*N* = 1156, %)	HER2-null (62, 5.4%)	HER2 ultra-low (320, 27.7%)	HER2-low (774, 66.9%)	P (HER2 status)	
					Null vs ultra-low	Ultra-low vs low
Age/years						
Median/range	52/24–89	53/33–79	51/24–85	52/25–87	0.564*	0.511*
Mean ± SD	53.1 ± 11.3	53.7 ± 11.4	52.8 ± 11.1	53.3 ± 11.4		
Menopaual status						
Pre/perimenopausal	656 (56.7)	32 (51.6)	185 (57.9)	439 (56.7)	0.367	0.739
Postmenopausal	500 (43.3)	30 (48.4)	135 (42.1)	335 (43.3)		
Breast position						
Left	581 (50.3)	29 (46.8)	161 (50.3)	391 (50.5)	0.610	0.951
Right	575 (49.7)	33 (53.2)	159 (49.7)	383 (49.5)		
Tumor size/cm						
Median/range	2.0/0.3–9	2.0/0.6–5.5	1.9/0.4–6.5	2/0.3–9	0.527*	0.523*
Mean ± SD	2.1 ± 1.0	2.2 ± 1.0	2.1 ± 1.0	2.1 ± 1.1		
Tumor multiplicity						
Single	1050 (90.8)	55 (88.7)	293 (91.6)	702 (90.7)	0.470	0.650
Multiple	106 (9.2)	7 (11.3)	27 (8.4)	72 (9.3)		
Histologic type						
Ductal	984 (85.1)	44 (71.0)	270 (84.4)	670 (86.6)	**0.004**	0.638
Lobular	59 (5.1)	10 (16.1)	16 (5.0)	33 (4.3)		
Other	113 (9.8)	8 (12.9)	34 (10.6)	71 (9.1)		
Grade						
1	80 (6.9)	7 (11.3)	26 (8.1)	47 (6.1)	0.156	0.389
2	891 (77.1)	41 (66.1)	248 (77.5)	602 (77.8)		
3	185 (16.0)	14 (22.6)	46 (14.4)	125 (16.1)		
pT						
1	659 (57.0)	33 (53.2)	189 (59.1)	437 (56.5)	0.668	0.716
2	473 (41.0)	28 (45.2)	125 (39.1)	320 (41.3)		
3	24 (2.0)	1 (1.6)	6 (1.8)	17 (2.2)		
pN						
0	143 (12.4)	8 (12.9)	37 (11.6)	98 (12.7)	0.102	0.211
1	240 (20.8)	10 (16.1)	56 (17,5)	174 (22.5)		
2	78 (6.7)	9 (14.5)	17 (5.3)	52 (6.7)		
3	42 (3.6)	1 (1.6)	12 (3.7)	29 (3.7)		
Unknown	653 (56.5)	34 (54.9)	198 (61.9)	421 (54.4)		
ER expression						
Median/range	90/1–100	90/1–95	90/1–100	90/1–100	0.083*	0.036*
Mean ± SD	79.5 ± 21.9	72.0 ± 28.5	77.8 ± 23.7	80.8 ± 20.3		
Ki67						
Median/range	2/1–90	2/1–80	2/1–90	2/1–80	0.519	<0.001*
Mean ± SD	9.4 ± 15.3	10.6 ± 18.1	12.3 ± 18.0	8.1 ± 13.6		
TILs						
Median/range	5/1–90	5/1–70	5/1–80	5/1–90	0.618*	0.035*
Mean ± SD	12.0 ± 13.1	11.6 ± 15.0	10.7 ± 11.9	12.5 ± 13.4		
Unknown	15	2	6	7		
Type of surgery						
BCS/+BR	292 (25.3)	11 (17.7)	85 (26.6)	196 (25.3)	0.341	0.121
Mastectomy	349 (30.2)	23 (37.1)	107 (33.4)	219 (28.3)		
Mastectomy + ALND	515 (44.5)	28 (45.2)	128 (40.0)	359 (46.4)		
Recurrence or metastasis						
Yes	39 (3.4)	3 (4.8)	11 (3.4)	26 (3.4)	0.483	0.948
No	1117 (96.6)	59 (95.2)	309 (96.6)	748 (96.6)		
Postoperative chemotherapy						
Yes	649 (56.1)	38 (61.3)	179 (55.9)	432 (55.8)	0.216	0.725
No	498 (43.1)	22 (35.5)	138 (43.1)	338 (43.7)		
Unknown	9 (0.8)	2 (3.2)	3 (1.0)	4 (0.5)		
Postoperative radiotherapy						
Yes	242 (21.0)	15 (24.2)	58 (18.1)	169 (21.8)	0.509	0.083
No	897 (77.6)	46 (74.2)	254 (79.4)	597 (77.1)		
Unknown	17 (1.4)	1 (1.6)	8 (2.5)	8 (1.1)		
Postoperative endocrine therapy						
Yes	980 (84.8)	49 (79.0)	273 (85.3)	658 (85.0)	0.094	0.951
No	146 (12.6)	13 (21.0)	39 (12.2)	94 (12.2)		
Unknown	30 (2.6)	0 (0.0)	8 (2.5)	22 (2.8)		

*BC* breast cancer; *HR* hormone receptor; *ER* estrogen receptor; *TILS* tumor infiltrating lymphocytes; *BCS* breast-conserving surgery; *BR* breast reconstruction; *ALND* axillary lymph node dissection; *SD* standard deviation

Bold indicates *P* < 0.05

*T-test; other testing methods are Chi-square test or Fisher’s exact test

**Table 3:** Baseline clinicopathological characteristics according to HER2 status of 207 HR− BC patients

Clinicopathological characteristics	Overall (*N* = 207, %)	HER2-null (24, 11.6%)	HER2 (75, 36.2%)	HER2-low (108, 52.2%)	P (HER2 status)	
					Null vs ultra-low	Ultra-low vs low
Age/years						
Median/range	54/28–84	52/30–76	52/28–78	55/31–84	0.828*	0.013*
Mean ± SD	53.2 ± 10.9	50.1 ± 11.3	51.2 ± 11.0	55.2 ± 10.5		
Menopaual status						
Pre/perimenopausal	115 (55.6)	15 (62.5)	47 (62.7)	53 (49.1)	0.988	0.069
Postmenopausal	92 (44.4)	9 (37.5)	28 (37.3)	55 (50.9)		
Breast position						
Left	115 (55.6)	15 (62.5)	45 (60.0)	55 (50.9)	0.827	0.225
Right	92 (44.4)	9 (37.5)	30 (40.0)	53 (49.1)		
Tumor size/cm						
Median/range	2.3/0.3–10	2.0/0.8–3.3	2.2/0.5–7.5	2.5/0.3–10	0.176*	0.121*
Mean ± SD	2.5 ± 1.2	2.0 ± 0.5	2.4 ± 1.1	2.6 ± 1.3		
Tumor multiplicity						
Single	190 (91.8)	24 (1.0)	68 (90.7)	98 (90.7)	0.190	0.986
Multiple	17 (8.2)	0 (0.0)	7 (9.3)	10 (9.3)		
Histologic type						
Ductal	161 (77.8)	19 (79.2)	61 (81.3)	81 (75.0)	0.797	0.556
Lobular	4 (1.9)	0 (0.0)	1 (1.3)	3 (2.8)		
Other	42 (20.3)	5 (20.8)	13 (17.4)	24 (22.2)		
Grade						
1	1 (0.5)	0 (0.0)	0 (0.0)	1 (0.9)	0.804	**0.035**
2	38 (18.4)	3 (12.5)	8 (10.7)	27 (25.0)		
3	168 (81.1)	21 (87.5)	61 (81.3)	80 (74.1)		
pT						
1	87 (42.0)	14 (58.3)	35 (46.7)	38 (35.2)	0.486	0.270
2	113 (54.6)	10 (41.7)	38 (50.7)	65 (60.2)		
3	7 (3.4)	0 (0.0)	2 (2.6)	5 (4.6)		
pN						
0	41 (19.8)	5 (20.8)	17 (22.7)	19 (17.6)	0.985	0.266
1	25 (12.1)	2 (8.3)	6 (8.0)	17 (15.7)		
2	7 (3.4)	0 (0.0)	1 (1.3)	6 (5.6)		
3	9 (4.3)	1 (4.2)	3 (4.0)	5 (4.6)		
Unknown	125 (60.4)	16 (66.7)	48 (64.0)	61 (56.5)		
Ki67						
Median/range	2/1–90	16/1–90	2/1–90	2/1–80	0.791*	0.003*
Mean ± SD	23.1 ± 30.0	31.8 ± 32.6	30.0 ± 32.3	16.7 ± 25.9		
TILs						
Median/range	15/1–90	10/1–80	20/1–90	15/1–90	0.488*	0.786*
Mean ± SD	22.7 ± 20.9	20.0 ± 25.8	23.5 ± 20.8	22.7 ± 20.0		
Unknown	3	0	0	3		
Type of surgery						
BCS/+BR	51 (24.6)	8 (33.3)	19 (25.4)	24 (22.2)	0.746	0.408
Mastectomy	69 (33.4)	8 (33.3)	28 (37.3)	33 (30.6)		
Mastectomy + ALND	87 (42.0)	8 (33.3)	28 (37.3)	51 (47.2)		
Recurrence or metastasis						
Yes	16 (7.7)	3 (12.5)	2 (2.7)	10 (9.3)	0.090	0.126
No	191 (92.3)	21 (87.5)	73 (97.3)	98 (90.7)		
Postoperative chemotherapy						
Yes	198 (95.7)	23 (95.8)	72 (96.0)	103 (95.4)	1.000	1.000
No	9 (4.3)	1 (4.2)	3 (4.0)	5 (4.6)		
Postoperative radiotherapy						
Yes	34 (16.4)	4 (16.7)	12 (16.0)	18 (16,7)	1.000	0.905
No	173 (83.6)	20 (83.3)	63 (84.0)	63 (58.3)		

*BC* breast cancer; *HR *hormone receptor; *TILS* tumor infiltrating lymphocytes; *BCS* breast-conserving surgery; *BR* breast reconstruction; *ALND* axillary lymph node dissection; *SD* standard deviation

Bold indicates *P* < 0.05

*T-test; other testing methods are Chi-square test or Fisher’s exact test

### Survival and prognosis

**Fig. 1:**
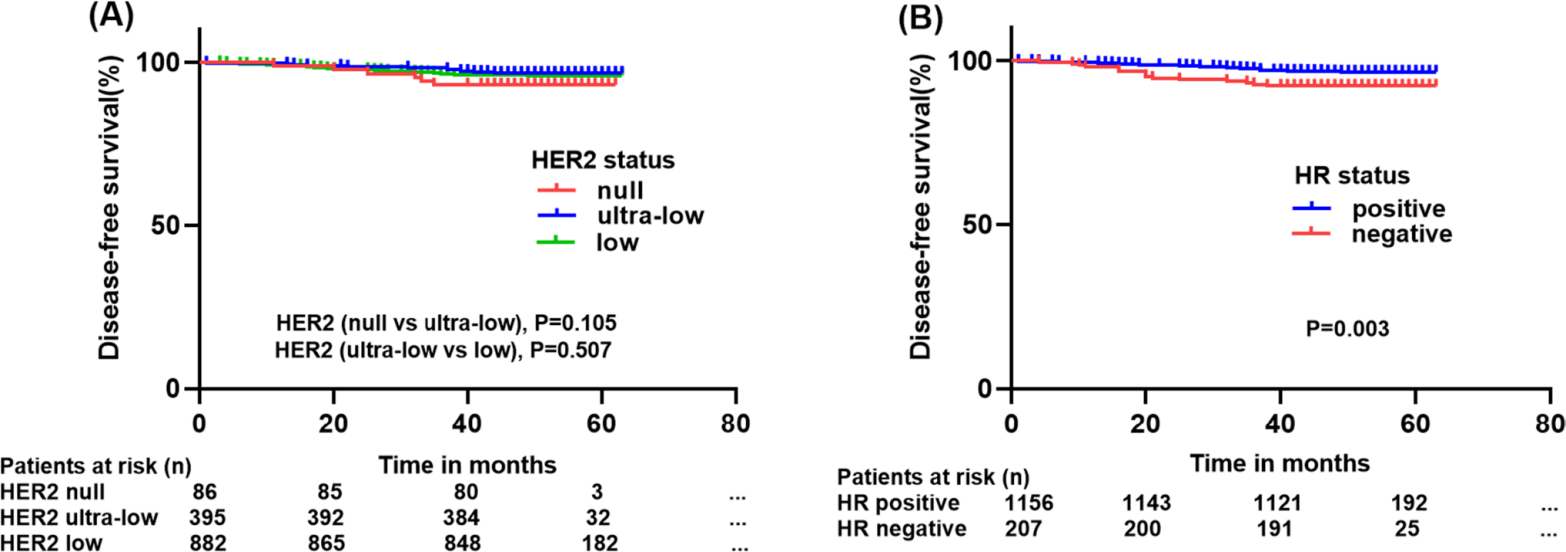
**A** Kaplan–Meier curve of disease-free survival (DFS) according to HER2 status (HER2 null vs HER2 ultra-low, *P* = 0.105; HER2 ultra-low vs HER2 low, *P* = 0.507, log–rank) in overall breast cancer (BC) patients; **B** Kaplan–Meier curve of DFS according to hormone receptor (HR) status (*P* = 0.003) in overall BC patients

In the survival analysis, DFS was not significantly different in HER2 ultra-low patients compared with HER2-null and low patients (HER2-null vs HER2 ultra-low, *P* = 0.105; HER2 ultra-low vs HER2-low, *P* = 0.507, log–rank). However, HR-positive patients had better DFS than HR-negative patients regardless of HER2 status (log–rank, *P* = 0.003). The Kaplan–Meier survival curve is shown in Fig. 1.

In the univariate and multivariate cox regression analysis of DFS in BC patients **(**Table 4**)**, we performed univariate analysis for all clinical variables and included meaningful ones in the multivariate analyses. The univariate analysis revealed that age (*P* = 0.048), tumor size (*P* = 0.013), histologic grade (*P* = 0.003), HR status (*P* = 0.004), and postoperative endocrine treatment (*P* < 0.001) were prognostic factors affecting DFS in patients with HER2-negative patients, and multivariate analysis found age (HR [95% CI] = 0.950 [0.928, 0.972], *P* < 0.001), HR status (HR [95% CI] = 3.342 [1.658, 6.736], *P* = 0.001), and postoperative endocrine therapy (HR [95% CI] = 0.048 [0.048, 0.023], *P* < 0.001) were independent prognostic factors influencing DFS in HER2-negative patients.

**Table 4:** Univariate and multivariate Cox regression model for DFS in overall BC patients

	Univariate analysis		Multivariate Cox regression model*	
	HR (95% CI)	*P*	HR (95% CI)	*P*
Age/years (24–89)	0.975 (0.952–1.000)	0.048	0.950 (0.928–0.972)	<0.001
Tumor size/cm (0.3–10)	1.261(1.049–1.514)	0.013	ns	ns
Grade 1		Ref		Ref
2	ns	ns	ns	ns
3	0.443 (0.260–0.753)	0.003	ns	ns
Hormone receptor status (− vs +)	0.423 (0.237–0.758)	0.004	3.342 (1.658–6.736)	0.001
Postoperative endocrine therapy (− vs +)	0.117 (0.064–0.214)	<0.001	0.048 (0.048–0.023)	<0.001

*BC* breast cancer; *DFS* disease-free survival; *HR* hazard ratio; *95% CI* 95% confidence interval; *Ref* reference; *ns* not statistically significant

^*^Variables that were significant in univariate analysis (*P* < 0.05) were included in multivariate Cox analysis, and other non-significant clinical parameters not shown

## Discussion

The development of novel HER2-directed ADCs [12] in the treatment of breast cancer with low expression of HER2 has dramatically changed the biologic and clinical treatment landscapes of HER2-negative BCs, and has been the driving force of the introduction of “HER2-low” subtype in BC. Currently, little is known regarding whether there are any differences between HER2-null, HER2 ultra-low and HER2-low BCs. In this study of 1363 HER2-negative BCs, we investigated the clinicopathological features and prognosis between HER2 ultra-low, HER2-null, and HER2-low expression in Chinese BC patients. Our results demonstrated that HER2 ultra-low BC patients had distinct clinicopathological features from HER2-null and HER2-low tumors; while, HER2 status (null, ultra-low, or low) had no prognostic value in these HER2-negative BC population. Consistent with the published literature [13], HR status was an independent prognostic factor for DFS in HER2-negative BC patients. To the best of our knowledge, this is by far the first study that focused on the group of HER2 ultra-low BC.

The clinicopathological features of BC patients with HER2-low expression (HER2 IHC 1+, and IHC 2+/ISH−) have been extensively studied in the past 2 years. For example, in a study [14] of 523 Chinese women with BC, Ning Liao et al. demonstrated that HER2-low breast tumors were enriched with HR-positive tumors, and who had lower Ki67 expression levels. In a study [15] published in the Lancet Oncology, Denkert et al. found that BC patients with low expression of HER2 were significantly different from those with zero expression of HER2 in terms of clinicopathological features. Significant differences between HER2-zero and HER2-low tumors were detected for hormone receptor status, Ki-67 proliferation rate and grading. Small but significant differences between HER2-zero (*P* = 0.028) and HER2-low (*P* = 0.0031) patients were seen in histopathological type and nodal status, no differences were seen for T-stage and TILs density.

Currently, the upper limit of low HER2 expression is clear (HER2 2+/ISH−); however, the lower limit is still un-defined, and whether HER2 ultra-low patients benefit from T-Dxd is under investigation. The preliminary result from DAISY trial [11] showed that about 30% of patients with HER2-ultra low BC benefited from T-Dxd. In addition, the ongoing DESTINY-Breast06 clinical study, which included the HER2 ultra-low population will further help define the lower limit of HER2 expression which would benefit from T-Dxd. These clinical trials have brought BC with HER2 ultra-low expression into focus. Up to now, there is no study comparing the differences between HER2-null, HER2-ultra-low, and HER2-low. Our study was the first to investigate the clinicopathological features of HER2 ultra-low expression BC patients. In our study population, HER2 protein expression for HER2-null, ultra-low, and low (1+ and IHC2+/ISH−) BCs accounted for 6.3%, 29.0%, and 64.7% respectively. Consistently, our data showed the proportion of HR+ in HER2-negative tumors (1156 cases, 84.8%) was much higher than in HR− (207 cases, 15.2%), in line with the current understanding of HER2-negative BCs [16, 17]. The results from this study showed that HER2 ultra-low patients were different from HER2-low patients in terms of N stage, HR status, Ki-67, and type of surgery. Additionally, significant differences in histologic type and postoperative endocrine therapy were observed between HER2 ultra-low and HER2-null patients. These findings suggest that HER2 ultra-low BC has certain distinct clinicopathological features from both HER2-null and HER2-low groups. However, further studies are needed to validate these findings, especially given that only 86 HER2-null patients were included in this study.

HER2 is a prognostic indicator in the BC and a predictor of drug therapeutic effect [18]. Regarding the effect of HER2 ultra-low expression on survival outcomes, it initially appeared that HER2-low expression might have better DFS compared to HER2 ultra-low expression; however, after adjusting for multiple relevant prognostic factors in multivariate analysis, we found that this did not hold true. Kaplan–Meier survival analysis found no effect of HER2 status, null, ultra-low, or low expression on DFS for HER2-negative patients, regardless of HR status. Previous literature is inconsistent with respect to outcome between HER2-low and HER2-zero (including both HER2-null and HER2 ultra-low) BCs. Some studies reported no difference in DFS or overall survival (OS) between HER2-zero and HER2-low BC patients [17, 19–21]. The report of Tarantino et al. [17] included 5235 patients with stage I to stage III HER2-negative BC and showed no significant difference in the prognosis of patients with HER2-low and HER2-zero BC. Agostinetto et al. [22] performed an analysis of the PAM50 intrinsic subtype for the heterogeneous disease of HER2-low expressing BC and highlighted differences in the clinical outcomes of this subtype and HER2 zero expressing BC. In addition, in several studies, HER2-low tumors were found to have lower pathological complete response (pCR) but no significant difference in DFS in clinical outcomes compared to HER2-zero tumors [14, 15]. Therefore, the prognostic role of HER2-low expression remains controversial. Interestingly, previous literature showed that HER2-low/HR+ tumors presented better progression-free interval (PFI) and disease-free interval (DFI) compared to HER2-low/HR− BC, and it further underlines the heterogeneity existing within the HER2-low group [22]. The survival analysis in the current study revealed that HER2 status (null, ultra-low or low expression) had no effect on DFS in HER2-negative BC patients (HER2-null vs HER2 ultra-low, *P* = 0.105; HER2 ultra-low vs HER2-low, *P* = 0.507, log–rank). However, regardless of HER2 status, HR-positive patients had better DFS than HR-negative patients (*P* = 0.003, log–rank). Further analysis showed that age (*P* < 0.001), HR status (*P* = 0.011), postoperative endocrine therapy (*P* < 0.001) were independent prognostic factors influencing DFS in HER2-negative patients. These results further support the concept that the clinical outcome is strongly associated with HR status, instead of HER2 expression level in the HER2-negative BCs.

Our study has several limitations. First of all, this was a retrospective single-center study and some imbalances between groups bias might exist. However, the intra-laboratory heterogeneity of HER2 detection was largely avoided. Secondly, our study did not include genomic information of HER2 ultra-low patients. Large-scale genomic analyses might shed some light on the genomic background of HER2 ultra-low patients in the future, meanwhile, there are very few studies on HER2 ultra-low expression breast cancer, so we need more prospective studies to support our findings.

In summary, this is by far the first study to investigate the clinicopathological features and outcome of HER2-negative BCs with focusing on the HER2-ultra low breast cancers. The results from this relative large-scale study showed that HER2 ultra-low BC patients in the Chinese population demonstrated distinct clinicopathological features from HER2-null and HER2-low tumors; while, HER2 status (null, ultra-low, or low) had no prognostic value in these HER2-negative BC population. Consistent with the published literature, HR status was an independent prognostic factor for DFS in HER2-negative BC patient. These results would increase our understanding of HER2 ultra-low BCs and support the concept that the clinical outcome is strongly associated with HR status, instead of HER2 expression level in the HER2-negative BCs.

## Data Availability

Data are available upon reasonable request to the authors.
